# A phase 2 randomized controlled dose-ranging trial of recombinant pertussis booster vaccines containing genetically inactivated pertussis toxin in women of childbearing age

**DOI:** 10.1016/j.vaccine.2021.10.076

**Published:** 2022-04-01

**Authors:** Kulkanya Chokephaibulkit, Thanyawee Puthanakit, Niranjan Bhat, Souad Mansouri, Yuxiao Tang, Keswadee Lapphra, Supattra Rungmaitree, Suvaporn Anugulruengkitt, Watsamon Jantarabenjakul, Indah Andi-Lolo, Renee Holt, Librada Fortuna, Chawanee Kerdsomboon, Pailinrut Chinwangso, Ladda Suwitruengrit, Anita H.J. van den Biggelaar, Simonetta Viviani, Hong Thai Pham, Bruce L. Innis

**Affiliations:** a Siriraj Institute of Clinical Research (SICRES), Faculty of Medicine Siriraj Hospital, Mahidol University, 2 Wanglang Road, Bangkok 10700, Thailand; b Department of Pediatrics, Faculty of Medicine Siriraj Hospital, Mahidol University, 2 Wanglang Road, 2 Wanglang Road, Bangkok 10700, Thailand; c Department of Pediatric, Faculty of Medicine and Center of Excellence in Pediatric Infectious Diseases and Vaccines, Chulalongkorn University, Rama IV Road, Bangkok 10330, Thailand; d Center for Vaccine Innovation and Access, PATH, 455 Massachusetts Avenue NW, Suite 1000, Washington, DC 20001, USA; e BioNet-Asia Co., Ltd., 19 Soi Udomsuk 37, Sukhumvit 103 Road, Bangjak, Prakanong, Bangkok 10260, Thailand

**Keywords:** Pertussis, Genetically-inactivated, Low-dose, Non-pregnant, Safety, Immunogenicity, PT_gen_, genetically-inactivated pertussis toxin, PT_chem_, chemically-inactivated pertussis toxin, ap1_gen_, monovalent recombinant acellular pertussis vaccine containing 1 μg PT_gen_ and 1 μg FHA, aP5_gen_, monovalent recombinant acellular pertussis vaccine containing 5 μg PT_gen_ and 5 µg FHA, Tdap1_gen_, tetanus, reduced-dose diphtheria, recombinant acellular pertussis vaccine containing 1 μg PT_gen_ and 1 μg FHA, Tdap2_gen_, tetanus, reduced-dose diphtheria, recombinant acellular pertussis vaccine containing 2 μg PT_gen_ and 5 µg FHA, TdaP5_gen_, tetanus, reduced-dose diphtheria, recombinant acellular pertussis vaccine containing 5 μg PT_gen_ and 5 µg FHA, Tdap8_chem_, tetanus, reduced-dose diphtheria, acellular pertussis vaccine containing 8 μg PT_chem_, 8 μg FHA and 2.5 μg pertactin (PRN)

## Abstract

**Background:**

A phase 2 randomized-controlled safety and immunogenicity trial evaluating different doses of recombinant acellular pertussis vaccine containing genetically-inactivated pertussis toxin (PT_gen_) was conducted in women of childbearing age in Thailand to identify formulations to advance to a trial in pregnant women.

**Methods:**

A total of 250 women were randomized 1:1:1:1:1 to receive one dose of one of three investigational vaccines including low-dose recombinant pertussis-only vaccine containing 1 μg PT_gen_ and 1 μg FHA (ap1_gen_), tetanus, reduced-dose diphtheria (Td) combined to ap1_gen_ (Tdap1_gen_) or combined to recombinant pertussis containing 2 μg PT_gen_ and 5 μg FHA (Tdap2_gen_), or one dose of licensed recombinant TdaP vaccine containing 5 μg PT_gen_ and 5 μg FHA (Boostagen®, TdaP5_gen_) or licensed Tdap vaccine containing 8 μg of chemically inactivated pertussis toxoid (PT_chem_), 8 μg FHA, and 2.5 μg pertactin (PRN) (Boostrix^TM^, Tdap8_chem_). Serum Immunoglobulin G (IgG) antibodies against vaccine antigens were measured before and 28 days after vaccination by ELISA. To advance to a trial in pregnant women, formulations had to induce a PT-IgG seroresponse rate with a 95% confidence interval (95% CI) lower limit of ≥ 50%.

**Results:**

Between 5 and 22 July 2018, a total of 250 women with median age of 31 years were enrolled. Post-vaccination PT-IgG seroresponse rates were 92% (95% CI 81–98) for ap1_gen_, 88% (95% CI 76–95) for Tdap1_gen_, 80% (95% CI 66–90) for Tdap2_gen_, 94% (95% CI 83–99) for TdaP5_gen_, and 78% (95% CI 64–88) for Tdap8_chem_. Frequencies of injection site and systemic reactions were comparable between the groups. No serious adverse events were reported during the 28-day post-vaccination period.

**Conclusions:**

All recombinant acellular pertussis vaccines were safe and immunogenic in women of childbearing age, and all met pre-defined immunogenicity criteria to advance to a trial in pregnant women.

Clinical Trial Registration: Thai Clinical Trial Registry, TCTR20180321004.

## Highlights

• Recombinant inactivated pertussis vaccines are safe in non-pregnant women.
• Lower-dose genetically-inactivated pertussis toxin has comparable immunogenicity.
• Monovalent recombinant pertussis vaccine shows favorable immunogenicity and safety.

## 1 Introduction

Respiratory infections, including pertussis, remain the main cause of death in children younger than 5 years globally [1], especially in infants who are too young to be protected by childhood vaccinations. The burden of pertussis is the highest in low- and middle-income countries (LMICs), but often goes unrecognized due to inadequate surveillance systems [2]. More than half of under-5 pertussis deaths occur in Africa, and another quarter in Southeast Asia [3], [4]. At the same time, circulation of pertussis in the community and consequently infection risk in newborns and young infants has increased in high-income countries (HICs) where exclusive vaccination with the less reactogenic acellular pertussis vaccines (aP) has contributed to relatively faster waning immunity and led to a rise in pertussis in all age groups including adolescents and adults [5].

In 2011, the US Advisory Committee on Immunization Practices (ACIP) recommended vaccination of pregnant women with pertussis vaccines to prevent pertussis deaths in very young infants [6]. In 2012, following an increase in pertussis-related hospitalizations and deaths in young infants, the United Kingdom (UK) implemented an emergency pertussis maternal immunization program [7]. Following evidence from the UK program that vaccination of pregnant women with pertussis vaccines is highly effective in preventing infant pertussis [8], [9], an increasing number of HICs have introduced maternal pertussis vaccination into their national immunization programs. Maternal pertussis immunization is now recognized as the most effective strategy to prevent severe pertussis disease and death in young infants [5]. Accordingly, the World Health Organization (WHO) is now recommending that countries with high infant pertussis morbidity and mortality rates consider implementing pertussis maternal immunization in the second or third trimester of pregnancy up to at least 15 days before delivery [10].

Pediatric vaccines containing diphtheria toxoid and tetanus toxoid combined to either whole cell pertussis (DTwP) or acellular pertussis (DTaP) vaccine are used for immunizing young children. Tdap formulations were developed for booster use in older children and adults and as indicated by the lowercase “d” and “p” contain lower concentrations of diphtheria toxoid and pertussis antigens to reduce the risk of adverse effects [11]. In the absence of a licensed pertussis-only vaccine (except for Thailand), adult Tdap formulation vaccines are used for maternal pertussis immunization [2]. However, in LMICs the uptake of maternal pertussis vaccines shall be only feasible if safe, effective, and affordable pertussis vaccines become available [2].

In the 1990s a pediatric recombinant pertussis vaccine containing only 5 µg of genetically-inactivated pertussis toxin (PT_gen_) (DTaP5_gen_) showed an efficacy similar to a pediatric vaccine containing 25 µg of chemically inactivated PT (PT_chem_) (DTaP25_chem_) [12]. More recently, other genetically-designed acellular pertussis vaccines also containing 5 µg of PT_gen_ in addition to 5 µg of *Bordetella pertussis* filamentous hemagglutin (FHA) were licensed in a monovalent formulation (aP5_gen_, Pertagen®) and in combination with tetanus and reduced-dose diphtheria toxoid (TdaP5_gen_, Boostagen®) for immunization of individuals aged 11 years and older in Thailand. Pre-licensure clinical trials demonstrated that both aP5_gen_ and TdaP5_gen_ are safe, and are more immunogenic and induce persisting higher antibody responses against pertussis toxin (PT) and FHA than chemically inactivated pertussis booster vaccines (Tdap_chem_) [13], [14], [15]. Subsequent follow-up studies demonstrated the persistence of pertussis antibody responses at significantly elevated levels 3 years after vaccination with aP5_gen_ and TdaP5_gen_ but not chemically inactivated pertussis booster vaccine [16].

PT-specific antibodies are considered to be important to protect young infants against severe pertussis disease. In a baboon model, infant monkeys born to mothers that were vaccinated with pertussis toxoid-only during pregnancy, were protected from disease following exposure to *B. pertussis*: this shows that anti-PT antibodies alone are sufficient to protect against pertussis disease [17].

Considering the high immunogenicity of PT_gen_, we postulated that lower-dose and therefore cost-saving formulations of the licensed recombinant pertussis vaccines may be suitable candidates for maternal pertussis immunization in LMICs if shown to induce pertussis antibody levels that are assumingly high enough to protect newborns against severe pertussis in the first months of life. To provide preliminary assurance of acceptable safety and immunogenicity before proceeding with a trial in pregnant women, we performed a randomized-controlled trial including lower-dose investigational formulations of these new generation recombinant pertussis vaccines in women of childbearing age.

## 2 Methods

### 2.1 Study design

This was a phase 2, dual-site, observer-blind, randomized, active-controlled vaccine trial evaluating in healthy women of childbearing age (18-40 years old) in Thailand, the immunogenicity and safety of a single intramuscular injection of a monovalent low-dose recombinant ap vaccine candidate (ap1_gen_, containing 1 μg PT_gen_ and 1 μg FHA), its tetanus, reduced-dose diphtheria combined formulation (Tdap1_gen_) and a combined reduced-dose recombinant ap vaccine candidate (Tdap2_gen_ containing 2 μg PT_gen_ and 5 µg FHA), including as reference vaccines licensed new generation recombinant TdaP5_gen_ vaccine (Boostagen®, containing 5 μg PT_gen_ and 5 µg FHA) and licensed Tdap_chem_ vaccine containing 8 μg of PT_chem_ and 8 μg FHA (Boostrix^TM^, Tdap8_chem_). The study was conducted according to International Conference on Harmonisation (ICH) and Good Clinical Practice guidelines, the Declaration of Helsinki and local ethical guidelines. Ethical approval was obtained from the Institutional Review Board, Faculty of Medicine Siriraj Hospital, Mahidol University, and Institutional Review Board, Faculty of Medicine, Chulalongkorn University. In line with Thai regulations, written informed consent was obtained from participants before recruitment into the study. This study is registered in the Thai Clinical Trial Registry (www.clinicaltrials.in.th), number TCTR20180321004.

### 2.2 Participants

Study participants were recruited at two sites in Bangkok, Thailand (250 participants; 125 per site): at the Faculty of Medicine Siriraj Hospital, Mahidol University, and at the Centre of Excellence in Pediatric Infectious Diseases and Vaccines, Chulalongkorn University. Inclusion criteria were: women aged 18–40 years (inclusive); no clinically significant health problems; a negative urine pregnancy test; willing to take birth control measures for 1 month after vaccination; willing and able to provide written informed consent; and comply with the study protocol. Main exclusion criteria were: pregnancy, breast-feeding or intending to become pregnant during the study period; history of significant medical illness including immune deficiency, uncontrolled diabetes or hypertension, heart or renal or hepatic diseases; a history of severe reactions to any vaccination or a history of severe allergic reactions; known hypersensitivity or allergy to diphtheria, tetanus, or pertussis vaccine (including excipients); receipt of any licensed vaccines within 30 days prior to enrolment (3 months for live attenuated vaccines) or plan to receive other vaccines during the study period; receipt of diphtheria or tetanus or pertussis vaccine within 1 year prior to enrolment; and history of any illness that, in the opinion of the investigator, might interfere with the results of the study or pose additional risk to the participants.

### 2.3 Vaccines

The composition of the study vaccines is shown in Table 1. The recombinant pertussis vaccines were developed and manufactured at BioNet-Asia, Ayutthaya, Thailand. The vaccines were administered by intramuscular injection preferably in the non-dominant deltoid region.

**Table 1:** Study vaccines

	Investigational vaccines			Licensed vaccines	
	Genetically-inactivated PT (PT_gen_) based vaccines			PT_gen_-based reference vaccine	Active comparator
	ap_gen_ low dose (ap1_gen_)^a^	Tdap_gen_ low dose (Tdap1_gen_)^a^	Tdap_gen_ reduced dose (Tdap2_gen_)^a^	TdaP_gen_ high dose (TdaP5_gen_; Boostagen®)^a,c^	Tdap_chem_ reduced dose (Tdap8_chem_; Boostrix™)^b,d^
**Active ingredients per 0.5-mL dose**					
**TT**	-	7.5 Lf	7.5 Lf	7.5 Lf	5 Lf
**DT**	-	2.0 Lf	2.0 Lf	2.0 Lf	2.5 Lf
**PT**	1 µg PT_gen_	1 µg PT_gen_	2 µg PT_gen_	5 µg PT_gen_	8 µg PT_chem_
**FHA**	1 µg	1 µg	5 µg	5 µg	8 µg
**PRN**	-	-	-	-	2.5 µg

a) Manufactured by BioNet-Asia Co., Ltd.; b) Manufactured by GlaxoSmithKline (GSK); c) Boostagen® and Boostrix^TM^ are licensed (reference) vaccines. Boostrix^TM^, containing chemically detoxified pertussis toxin, is included as the comparator to the other vaccines that all include genetically inactivated pertussis toxin. Abbreviations: TT, Tetanus Toxoid; DT, Diphtheria Toxoid; PT, Pertussis Toxoid; PT_gen_, genetically inactivated PT; PT_chem_, chemically inactivated PT; FHA, filamentous hemagglutinin; PRN, pertactin; Lf, limit of flocculation.

Acellular pertussis vaccines currently available from different manufacturers should be considered as different and unique products because of the presence of one or more different components, i.e. chemically (PT_chem_) or genetically detoxified pertussis toxin (PT_gen_), filamentous haemagglutinin (FHA), pertactin (PRN), and fimbriae (FIM). In this study we differentiate “component” and “valence” as follows: the recombinant acellular pertussis vaccines studied here contain two components (PT_gen_ and FHA), whereas the chemically detoxified acellular pertussis comparator is a three-component vaccine containing PT_chem_, FHA and PRN; the study also evaluates a monovalent recombinant pertussis vaccine (pertussis-only) and trivalent (combination) vaccines containing tetanus, diphtheria in addition to pertussis vaccines (Tdap or TdaP, with lowercase ‘p’ referring to lower-dose pertussis, and uppercase ‘P’ to full-dose pertussis). For example, ap1_gen_ a two-component monovalent lower-dose ap vaccine.

### 2.4 Randomization and blinding

Randomization occurred in a 1:1:1:1:1 ratio per study site (50 subjects per vaccine group; 25 per study site). A randomization list was provided to non-blinded clinical research staff who were responsible for participant randomization, vaccine preparation, administration and accountability. All other research staff and study participants were blinded to the vaccine administered. Participants assigned to receiving ap1_gen_ were unblinded 28 days after vaccination and offered a Td vaccine after the second blood collection.

### 2.5 Immunogenicity assessment

Venous blood samples (5 mL) were obtained on the same day before vaccine administration (Day 0; baseline) and 28 days after vaccination (Day 28), and processed for serum separation. Serum Immunoglobulin G (IgG) antibody titers against PTgen and FHA were measured, using validated indirect enzyme-linked immunosorbent assays (ELISA) at BioNet-Asia’s Human Serology Laboratory in Ayutthaya, Thailand [18]. Concentrations were expressed in IU/mL whilst calibrated to the WHO International Standard Pertussis Antiserum (Human) 06/140. The WHO Reference Reagent Pertussis Antiserum (Human) 06/142 (NIBSC, UK) was used as positive control. The lower limit of quantification (LLOQ) was below the assay cut-off of 5 IU/mL for PT-IgG, and 1 IU/mL for FHA-IgG. Seroresponsiveness was defined according to criteria used for approval of Boostrix^TM^ for adult vaccination in the US, namely [19]: in participants with pre-vaccination antibody concentrations titres < 5 IU/mL, post-vaccination antibody concentrations ≥ 20 IU/mL; in participants with pre-vaccination antibody concentrations ≥ 5 IU/mL and < 20 IU/mL, an increase of at least 4 times the pre-vaccination concentration; in participants with pre-vaccination antibody concentrations ≥ 20 IU/mL, an increase of at least 2 times the pre-vaccination antibody concentration.

Serum TT- and DT-specific IgG titers were measured using validated commercially available ELISA kits (Serion ELISA classic Diphtheria IgG kit, ESR130G; and Serion ELISA Classic Tetanus IgG kit, ESR108G, Virion/ Serion, Germany). Seroprotection rates for tetanus and diphtheria were defined based on TT-IgG and DT-IgG antibody titres ≥ 0.1 IU/mL.

### 2.6 Safety assessment

Participants were monitored for 30 min post-immunization for any immediate reactions. Diary cards were distributed on the day of vaccination to record solicited adverse events (AEs) at the injection site (pain, redness, swelling and induration), or systemic AEs (fever, headache, fatigue, malaise, arthralgia, chills, myalgia, nausea and vomiting) for 7 days after vaccination. Unsolicited AEs and serious adverse events (SAEs) were collected for the study duration of 28 days. AEs were assessed by the investigator for severity following the Division of AIDS (DAIDS) Table for Grading the Severity of Adult and Pediatric Adverse Events of the US National Institute of Health, 2017 [20], and causality to study vaccine according to ICH guidelines and protocol-specified safety considerations.

### 2.7 Outcomes

The aim was to identify formulations of recombinant acellular pertussis vaccine to be included in a maternal immunization trial, based on safety and a minimum level of immunogenicity against PT. To meet the criterion for inclusion in a maternal vaccination trial, a formulation had to induce a PT-IgG seroresponse rate with a 95% confidence interval (95% CI) lower limit equal to or greater than 50%.

### 2.8 Statistical analysis

Two hundred fifty (2 5 0) participants were planned to be enrolled in this proof-of-concept study based on clinical and practical considerations. All analyses for immunogenicity and safety endpoints were descriptive and no multiplicity adjustment was carried out. Data management and statistical analyses were performed by the Center of Excellence for Biomedical and Public Health Informatics (BIOPHICS), Bangkok, Thailand. All statistical analyses were conducted using Statistical Analysis System (SAS®) software version 9.4. Seroresponse rates for PT-IgG and FHA-IgG, and seroprotection rates for TT-IgG and DT-IgG were computed along with its corresponding exact two-sided 95% CI based on the Clopper-Pearson method for each vaccine group. Differences between the recombinant pertussis vaccine groups and licensed Tdap8_chem_ comparator were calculated along its two-sided 95% CI obtained by the Miettinen and Nurminen method. For safety analysis, the proportion of subjects with safety endpoints at different study time points along with an exact two-sided 95% CI were calculated using the Clopper-Pearson method.

## 3 Results

### 3.1 Study population

A total of 257 women of childbearing age were screened, of whom 250 (125 per study site) were enrolled and randomized into one of 5 vaccine groups (50 per treatment group) between July 5 to 22, 2018. The main reason for screening failures was having an acute or chronic clinically significant psychiatric, hematologic, pulmonary, cardiovascular, or hepatic or renal functional abnormality as determined by the Investigator based on medical history and physical examination. All 250 participants completed the study and were included in the safety and immunogenicity analyses. Demographic characteristics (age, height and body weight) at baseline were similar for participants in all vaccine groups (Table 2).

**Table 2:** Demographics of enrolled women of childbearing age at baseline.

	ap1_gen_ *(N = 50)*	Tdap1_gen_ *(N = 50)*	Tdap2_gen_ *(N = 50)*	TdaP5_gen_ *(N = 50)*	Tdap8_chem_ *(N = 50)*
**Age (years)** mean (sd) (min-max)	29.7 (5.3) (18-39)	29.5 (6.3) (18-39)	30.9 (4.7) (22-39)	30.3 (5.7) (18-39)	32.0 (5.2) (19-39)
**Height (cm)** mean (sd) (min-max)	158.5 (5.6) (147-173)	158.5 (4.5) (148-170)	157.9 (5.7) (144-174)	157.7 (5.2) (145-167)	159.3 (5.5) (145-175)
**Weight (kg)** mean (sd) (min-max)	57.7 (13.5) (39.3-96.1)	60.1 (13.7) (41.2-101.1)	57.9 (13.1) (38.8-95.9)	62.2 (16.4) (40.8-111.6)	63.8 (14.5) (42.6-101.1)

Abbreviations: sd, standard deviation; min, minimum; max, maximum.

### 3.2 Geometric mean concentrations (GMC) and GMC ratios of post- to pre-vaccination

**Fig. 1:**
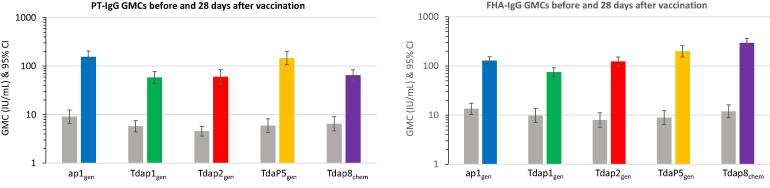
Geometric mean concentrations of PT-IgG and FHA-IgG antibodies before and after vaccination with recombinant pertussis vaccines and a chemically inactivated comparator pertussis vaccine in women of childbearing age. Bars represent GMC with 95% confidence intervals (95% CI). Baseline responses are presented in grey bars, and 28 days post-vaccination responses in coloured bars (ap1_gen_, blue; Tdap1_gen_, green; Tdap2_gen_, red; TdaP5_gen_, orange; Tdap8_chem_, purple).

All vaccines were immunogenic in this study population, as shown by significantly elevated PT-IgG and FHA-IgG antibodies 28 days after vaccination compared to baseline (Fig. 1 & Table 3). GMCRs for PT-IgG were significantly higher in the group that received the licensed recombinant TdaP5_gen_ compared to the licensed Tdap8_chem_ group, while GMCRs for FHA-IgG were comparable. The lower-dose recombinant pertussis vaccines induced PT-IgG responses resulting in GMCRs that were comparable to that induced by Tdap8_chem_, with the highest response induced by ap1_gen_ that only contains 1 µg of PT_gen_. Recombinant vaccines containing 5 µg of FHA (TdaP5_gen_ and Tdap2_gen_) induced FHA-IgG responses associated with GMCRs that were comparable to Tdap8_chem_ (containing 8 µg FHA). The low-dose recombinant ap1_gen_ and Tdap1_gen_ vaccines containing 1 µg of FHA only, resulted in FHA-IgG responses that were respectively 9.6-fold and 7.6-fold higher than baseline Table 3.

**Table 3:** Geometric mean concentration ratio (GMCR) of PT-IgG and FHA-IgG antibodies at 28 days after vaccination compared to baseline in different vaccine groups.

	GMC Ratio (95% CI)				
	ap1_gen_ *(N = 50)*	Tdap1_gen_ *(N = 50)*	Tdap2_gen_ *(N = 50)*	TdaP5_gen_ *(N = 50)*	Tdap8_chem_ *(N = 50)*
PT-IgG	17.2 (11.9-24.9)	10.1 (7.4-13.7)	13.3 (9.8-18.0)	24.7 (18.2-33.4)	9.9 (7.4-13.4)
FHA-IgG	9.6 (7.2-12.8)	7.6 (6.0-9.7)	15.6 (11.2-21.6)	22.2 (15.7-31.3)	24.6 (16.7-36.2)

Geometric mean concentration ratios (GMCRs) for PT-IgG and FHA-IgG antibody responses at Day 28 after vaccination compared to pre-vaccination were calculated for the different vaccine groups, and presented including their 95% confidence intervals (95% CI).

**Fig. 2:**
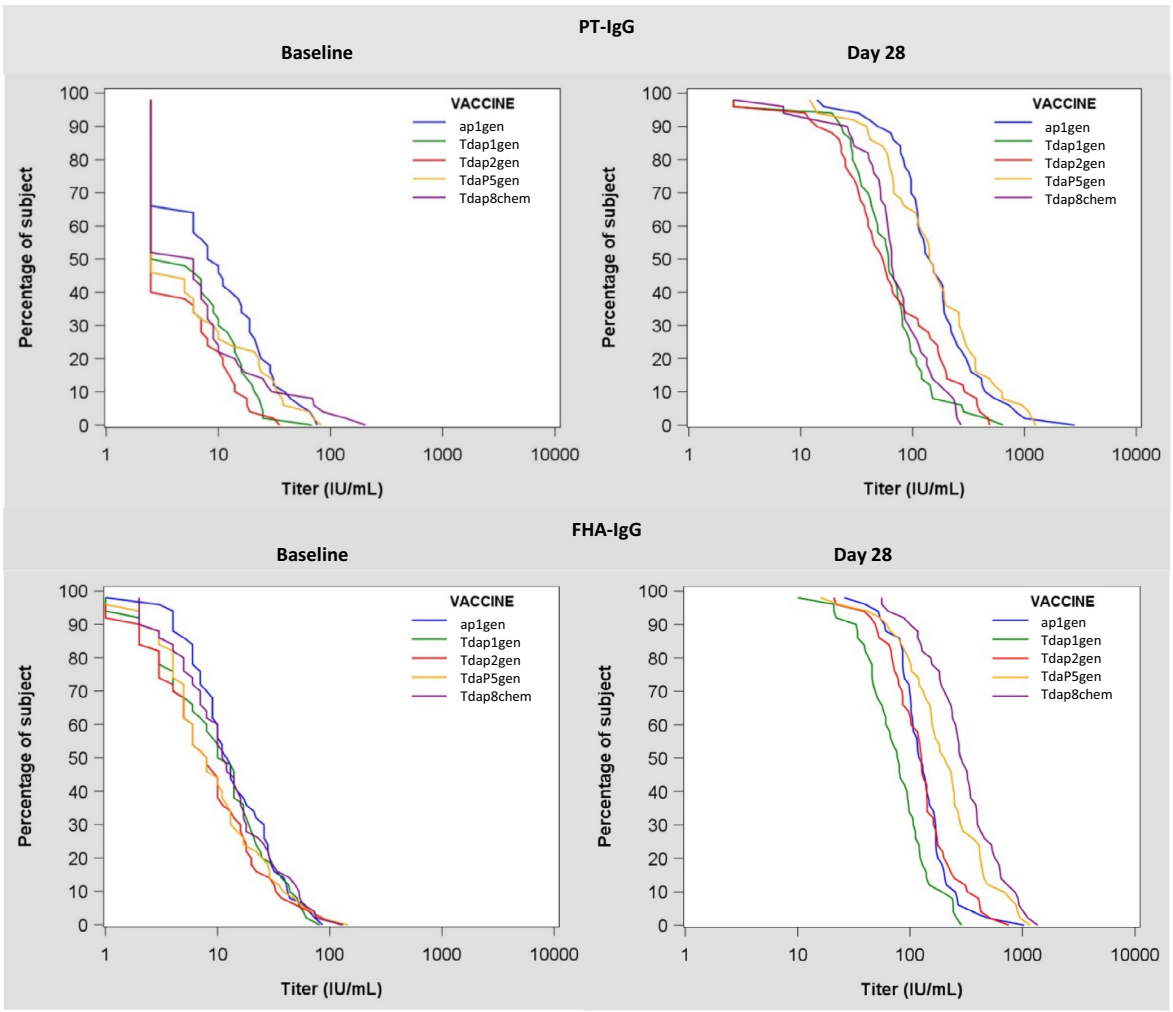
Reverse cumulative distribution curves (RCDCs) for PT-IgG and FHA-IgG antibody titers at baseline and 28 days post-vaccination with recombinant pertussis vaccines and a chemically inactivated comparator pertussis vaccine in women of childbearing age. (ap1_gen_, blue; Tdap1_gen_, green; Tdap2_gen_, red; TdaP5_gen_, orange; Tdap8_chem_, purple).

### 3.3 Seroresponse rates for pertussis antigens

PT-IgG seroresponse rates were highest in the licensed recombinant TdaP5_gen_ group (94.0%, 95% CI 83.5–98.8) and lowest in the comparator Tdap8_chem_ group (78.0%, 95% CI 64.0–88.5), with an observed 16.0% (95% CI 2.6–30.3) difference between the two (Table 4). Vaccination with recombinant ap1_gen_ resulted in a 92.0% (95% CI 80.8–97.8) seroresponse rate: a 14.0% (95% CI −0.1–28.6) difference from Tdap8_chem_.

Seroresponse rates for FHA-IgG were similar between the vaccine groups. Rates ranged from 90.0% (95% CI 78.2–96.7) in the ap1_gen_ group to 100% (95% CI 92.9–100) in the Tdap2_gen_ group (Table 4).

### 3.4 Reverse cumulative distribution curves (RCDCs)

RCDCs for PT-IgG antibody responses show that compared to other groups (i.e. Tdap1_gen_, Tdap2_gen_, and Tdap8_chem_), higher titers were achieved in relatively more individuals vaccinated with recombinant TdaP5_gen_ or ap1_gen_ (Fig. 2). For FHA-IgG, vaccination with licensed Tdap8_chem_ or TdaP5_gen_ vaccines resulted in overall higher antibody responses.

### 3.5 Seroprotection rates for tetanus and diphtheria

All participants except for two women in the Tdap1_gen_ group and one in the Tdap2_gen_ group, had protective TT-IgG antibody levels of ≥ 0.1 IU/mL before vaccination. All participants who received a Td-containing vaccine were seroprotected for tetanus 28 days after vaccination (Table 5).

**Table 4:** Seroresponse rate for PT-IgG and FHA-IgG antibodies at 28 days after vaccination compared to baseline, and difference in recombinant pertussis vaccine groups compared to comparator Tdap8_chem_.

	ap1_gen_ *(N = 50)*	Tdap1_gen_ *(N = 50)*	Tdap2_gen_ *(N = 50)*	TdaP5_gen_ *(N = 50)*	Tdap8_chem_ *(N = 50)*
**Seroresponse rate, PT-IgG**					
% (95% CI)	92.0% (80.8-97.8)	88.0% (75.7-95.5)	80.0% (66.3-90.0)	94.0% (83.5-98.8)	78.0% (64.0-88.5)
ΔTdap8_chem_ (95% CI)	14.0% (-0.1-28.6)	10.0% (-5.1-25.2)	2.0% (-14.3-18.3)	16.0% (2.6-30.3)	--
**Seroresponse rate, FHA-IgG**					
% (95% CI)	90.0% (78.2-96.7)	92.0% (80.8-97.8)	100% (92.9-100)	96.0% (86.3-99.5)	96.0% (86.3-99.5)
ΔTdap8_chem_ (95% CI)	-6.0% (-18.0-4.9)	-4.0% (-15.5-6.6)	4.0% (-3.4-13.5)	0% (-10.1-10.1)	--

Δ Tdap8_chem_ presents the difference in seroresponse rate for participants in the recombinant vaccine group minus that for participants in the comparator Tdap8_chem_ group. Seroresponse is defined as: for seronegative subjects at baseline (<5 IU/mL), post-vaccination antibody concentrations ≥20 IU/mL; for seropositive subjects at baseline with pre-vaccination antibody concentrations ≥5 IU/mL and <20 IU/mL, an increase of at least 4 times the pre-vaccination antibody concentrations; for seropositive subjects with pre-vaccination antibody concentrations ≥20 IU/mL, an increase of at least 2 times the pre-vaccination antibody concentrations. Abbreviations: CI, confidence interval; IU, international units.

Seroprotection rates for diphtheria before vaccination were similar between the groups, ranging from 60% to 70%. Seroprotection rates for DT-IgG increased and were comparable between the groups after vaccination with a diphtheria-containing vaccine, ranging from 94% to 100% (Table 5).

### 3.6 Solicited and unsolicited (serious) adverse events

Pain at the injection site was reported at similar rates in all vaccine groups as the most common solicited injection site reaction within 7 days after vaccination (Table 6): the lowest rate was reported in the ap1_gen_ group (70%), and the highest rate in the Tdap1_gen_ group (84%). Pain was mostly mild in severity and resolved within a few days. Severe pain was reported by 2% to 3% of participants vaccinated with a Td-containing vaccine. None of the participants in the ap1_gen_ group reported severe pain. Table 6.

**Table 5:** Seroprotection rates for tetanus and diphtheria before and 28 days after vaccination with Td combination vaccines containing recombinant pertussis vaccines or chemically inactivated pertussis vaccine.

Seroprotection	Tdap1_gen_ *(N = 50)*	Tdap2_gen_ *(N = 50)*	TdaP5_gen_ *(N = 50)*	Tdap8_chem_ *(N = 50)*
**TT-IgG Day 0**, % (95% CI)	96.0% (86.3-99.5)	98.0% (89.4-100)	100% (92.9-100)	100% (92.9-100)
**TT-IgG Day 28**, % (95% CI)	100% (92.9-100)	100% (92.9-100)	100% (92.9-100)	100% (92.9-100)
**DT-IgG Day 0**, % (95% CI)	60.0% (45.2-73.6)	66.0% (51.2-78.8)	64.0% (49.2-77.1)	70.0% (55.4-82.1)
**DT-IgG Day 28**, % (95% CI)	94.0% (83.5-98.8)	98.0% (89.4-100)	94.0% (83.5-98.8)	100% (92.9-100)

Seroprotection rates for tetanus and diphtheria were defined as TT-IgG or DT-IgG concentrations ≥0.1 IU/mL. Abbreviations: CI, confidence interval; IU, international units.

**Table 6:** Proportion of participants reporting solicited adverse events within 7 days after vaccination and unsolicited adverse events within 28 days after vaccination with recombinant pertussis and chemically inactivated pertussis vaccines.

	ap1_gen_ *(N = 50)*	Tdap1_gen_ *(N = 50)*	Tdap2_gen_ *(N = 50)*	TdaP5_gen_ *(N = 50)*	Tdap8_chem_ *(N = 50)*
**Local post-immunization reactions within 7 days**					
Pain, % (95% CI)	70.0% (55.4-82.1)	84.0% (70.9-92.8)	78.0% (64.0-88.5)	74.0% (59.7-85.4)	78.0% (64.0-88.5)
Redness, % (95% CI)	0.0% (0.0-7.1)	0.0% (0.0-7.1)	0.0% (0.0-7.1)	0.0% (0.0-7.1)	0.0% (0.0-7.1)
Swelling, % (95% CI)	0.0% (0.0-7.1)	2.0% (0.1-10.7)	2.0% (0.1-10.7)	0.0% (0.0-7.1)	0.0% (0.0-7.1)
Induration, % (95% CI)	0.0% (0.0-7.1)	2.0% (0.1-10.7)	0.0% (0.0-7.1)	2.0% (0.1-10.7)	0.0% (0.0-7.1)
**Systemic post-immunization reactions within 7 days**					
Headache, % (95% CI)	14.0 % (5.8-26.7)	24.0% (13.1-38.2)	20.0% (10.0-33.7)	26.0% (14.6-40.3)	26.0% (14.6-40.3)
Arthralgia, % (95% CI)	8.0% (2.2-19.2)	10.0% (3.3-21.8)	8.0% (2.2-19.2)	8.0% (2.2-19.2)	6.0% (1.3-16.6)
Chills, % (95% CI)	2.0% (0.1-10.7)	10.0% (3.3-21.8)	2.0% (0.1-10.7)	8.0% (2.2-19.2)	4.0% (0.5-13.7)
Fatigue, % (95% CI)	12.0% (4.5-24.3)	16.0% (7.2-29.1)	10.0% (3.3-21.8)	12.0% (4.5-24.3)	12.0% (4.5-24.3)
Malaise, % (95% CI)	12.0% (4.5-24.3)	16.0% (7.2-29.1)	16.0% (7.2-29.1)	18.0% (8.6-31.4)	6.0% (1.3-16.6)
Myalgia, % (95% CI)	40.0% (26.4-54.8)	38.0% (24.7-52.8)	42.0% (28.2-56.8)	42.0% (28.2-56.8)	42.0% (28.2-56.8
Fever ^a^, % (95% CI)	2.0% (0.1-10.7)	4.0% (0.5-13.7)	2.0% (0.1-10.7)	2.0% (0.1-10.7)	2.0% (0.1-10.7)
Vomiting, % (95% CI)	4.0% (0.5-13.7)	4.0% (0.5-13.7)	2.0% (0.1-10.7)	2.0% (0.1-10.7)	4.0% (0.5-13.7)
Nausea, % (95% CI)	4.0% (0.5-13.7)	4.0% (0.5-13.7)	4.0% (0.5-13.7)	8.0% (2.2-19.2)	8.0% (2.2-19.2)
**Unsolicited adverse events within 28 days**					
≥ 1 AE, % (95% CI)	22.0% (11.5-36.0)	26.0% (14.6-40.3)	24.0% (13.1-38.2)	20.0% (10.0-33.7)	26.0% (14.6-40.3)
≥ 1 vaccine related AE, % (95% CI)	6.0% (1.3-16.6)	16.0% (7.2-29.1)	10.0% (3.3-21.8)	6.0% (1.3-16.6)	12.0% (4.5-24.3)
Discontinued due to AE, % (95% CI)	0.0% (0.0-7.1)	0.0% (0.0-7.1)	0.0% (0.0-7.1)	0.0% (0.0-7.1)	0.0% (0.0-7.1)
≥ 1 SAE, % (95% CI)	0.0% (0.0-7.1)	0.0% (0.0-7.1)	0.0% (0.0-7.1)	0.0% (0.0-7.1)	0.0% (0.0-7.1)

a) Fever is defined as ≥38°C. Abbreviations: AE, adverse event; SAE, serious adverse event CI, confidence interval; IU, international units.

Rates of reported systemic post-immunization reactions were comparable between the groups (Table 6) and were mostly mild to moderate in severity and resolved in a few days without sequelae. Myalgia was the most common solicited systemic post-immunization reaction within 7 days after vaccination, and was reported by 38% of participants in the Tdap1_gen_ group, 40% in the ap1_gen_ group, and 42% of participants in each of the Tdap2_gen_, TdaP5_gen_ and Tdap8_chem_ groups.

The proportion of participants with one or more vaccine-related unsolicited AEs was comparable between the groups, varying between 6% and 16% (Table 6). Vaccine-related unsolicited AEs were mostly mild in severity and none led to hospitalization or discontinuation from the study. No SAEs were reported during the 28-day post-vaccination period.

## 4 Discussion

This phase 2 trial demonstrated that a licensed formulation of a new generation recombinant acellular pertussis vaccine (Boostagen®; TdaP5_gen_) and investigational formulations containing lower concentrations of PT_gen_, were safe and immunogenic in women of childbearing age. All formulations of recombinant pertussis vaccines that were tested induced a seroresponse rate between 80% and 92%: all with corresponding lower limit of 95% CIs greater than 50%, which was defined as the immunogenicity criterion for vaccine formulations to be included in a trial in pregnant women. This criterion was based on seroresponse rates as defined for licensure of Boostrix™ (the 3-component Tdap8_chem_ comparator used in this study) in adults in the US [19]. All recombinant vaccine formulations therefore met the pre-defined criterion for advancement to a subsequent maternal immunization trial.

Maternal immunization with 3- and 5-component Tdap_chem_ vaccines provides high levels of protection in infants in the first two months of life when they are the most vulnerable, with vaccine effectiveness ranging from 69% to 91% for pertussis prevention, from 91% to 94% for prevention of hospitalization, and 95% for prevention of death due to pertussis [21]. BioNet’s new generation monovalent recombinant 2-component acellular pertussis aP5_gen_ vaccine (Pertagen®) and trivalent (combination)  2-component acellular pertussis TdaP5_gen_ vaccine were earlier evaluated against the trivalent (combination) 5-component acellular pertussis Tdap_chem_ (Adacel®) in adolescents [13]. BioNet's monovalent aP5_gen_ vaccine was earlier also compared to Boostrix™ (trivalent, 3-component acellular pertussis Tdap8_chem_) in Swiss adolescents [15]. The phase 2 trial in women of reproductive age reported on here is the first study comparing TdaP5_gen_ to 3-component Tdap8_chem_.

PT-IgG seroresponse rates in women of childbearing age varied between 80% and 94% for all tested recombinant pertussis vaccine formulations. The second highest seroresponse rate (92%) was found for recombinant ap1_gen_ that contains only 1 µg of PT_gen_ while Tdap8_chem_ that contains 8 µg of chemically inactivated PT was associated with a 78% response rate, which is in line with reports by others [19]_._ This supports earlier reports from us and others that genetically inactivated PT is more immunogenic than PT that is chemically inactivated, and which is explained by the preservation of the toxin’s native epitope structure and immunogenic properties in genetic inactivation [12], [13], [22]. Moreover, pertussis-only vaccine may also be more immunogenic than combination (multivalent) vaccines because of the absence of concomitantly administered vaccine antigens (i.e. diphtheria and tetanus) that could potentially interfer with the pertussis response [23], and that for the same reason may also be less reactogenic as supported in this study by the high immunogencity and low reactogenicity of ap1_gen_.

In TdaP5_gen_ licensure-enabling and subsequent follow-up studies, the immunogenicity and persistence of antibody responses against FHA were demonstrated to remain higher in adolescents vaccinated with a booster dose of the recombinant pertussis vaccine than those who had received the comparator Tdap vaccine containing the same dosage of FHA [13], [14], [16]. The likely explanation for this is that in chemically detoxified PT containing pertussis vaccines, FHA needs to be treated with high concentrations of formaldehyde sufficient to inactivate any residual PT co-eluted with FHA. This chemical treatment is known to affect the protein conformation and immune recognition of FHA, and hence will affect the immunogenicity of FHA [24]. In the recombinant pertussis vaccine, FHA is co-eluted with non-toxic genetically-detoxified PT and therefore is not exposed to chemical inactivation processes, resulting in retaining the functional and immunological properties of FHA.

In this study, the safety and tolerability of tested recombinant vaccines including licensed TdaP5_gen_ and all lower-dose formulations were similar to that of Tdap8_chem_. The lowest rate of solicited AEs occurred in the ap1_gen_ group, and this was the only group with no report of severe pain. This low-dose recombinant pertussis-only vaccine therefore is highly immunogenic and shows a favourable reactogenicity profile. This may offer an advantageous alternative for vaccinating pregnant women against pertussis when maternal diphteria and tetanus vaccinations are not required [25]. Vaccination during pregnancy is not necessary to protect neonatal infants against diphtheria if the mother has received a total of 6 doses of diphtheria toxoid vaccine (3-dose primary series in early childhood and 3 booster doses during childhood and adolescence) [26]. Pregnant women and their newborn infants are also protected from birth-associated tetanus if the mother received 6 doses of tetanus toxoid vaccine (3-dose primary series in early childhood and 3 booster doses preferably administered during childhood and completed by adolescence), or 5 doses in total if first vaccinated during adolescence/adulthood [27]. Although booster vaccination during pregnancy boosts immunity in women who have received the full set of recommended doses, there is no evidence to support the benefit of adult Td booster vaccinations beyond the recommended doses [28]. Notably, in countries where maternal-neonatal-tetanus remains a public health problem, pregnant women who cannot provide reliable information on previous tetanus vaccination, should receive at least 2 doses of tetanus toxoid vaccine, preferably in combination with diphtheria toxoid [27] and possibly pertussis toxoid.

The national immunization program in Thailand does not include pertussis booster vaccinations after pre-school age [29]. In our study at least 50% of women were seronegative for PT-IgG at baseline. This corresponds with findings from a 2014 seroprevalence study that reported between 43% and 51% of adults in Thailand were seronegative [30]. Another study found 67% of pregnant women in Thailand were seronegative [31]. These low PT-IgG levels in adults suggest that a booster dose of pertussis vaccine is required to address the waning immunity in adults. Pertussis booster vaccination would in particular be important in pregnant women to induce sufficiently high titers of PT-IgG to protect newborns against severe pertussis disease [17], [32].

In line with WHO recommendations several countries have introduced maternal pertussis vaccination [8], [9], [33], [34], [35], [36]. In the absence of pertussis-only vaccine, Tdap combination vaccines are used. The only stand-alone pertussis vaccine currently available is a 2-component recombinant aP vaccine, aP5_gen_ (Pertagen®, containing 5 μg PT_gen_ and 5 μg FHA) that together with the Td-combined formulation tested in this trial (TdaP5_gen_; Boostagen®) are licensed in Thailand for immunization of adolescents and adults: both recombinant pertussis vaccines are used by pregnant women. Pharmacovigilance data from active post-marketing surveillance indicate that both formulations of recombinant aP5_gen_-based vaccine have an acceptable safety profile in pregnant women, and are not associated with vaccine-related adverse effect on pregnancy or newborn health outcomes [37]. Moreover, in an observational study of women vaccinated with aP5_gen_ and TdaP5_gen_ in the 2nd or 3rd trimester of their pregnancy [38], cord blood PT-IgG titers were demonstrated to be 31.6-fold (95% CI 18.9–53.0) (aP5_gen_) and 23.5-fold (95% CI 14.0–39.3) (TdaP5_gen_) higher compared to cord titers in women who had received a Td-only vaccine in pregnancy, reaching GMCs of 206.1 IU/mL (95% CI 164.3–258.6) (aP5_gen_) and 153.1 IU/mL (95% CI 129.1–181.5) (TdaP5_gen_). Cord FHA-IgG GMCs in the aP5_gen_ group (217.2 IU/mL, 95% CI 184.0–256.4) and TdaP5_gen_ group (232.0 IU/mL, 95% CI 199.0–270.6) were respectively 17.8-fold (95% CI 11.6–27.3) and 19.0-fold (95% CI 12.4–29.1) higher.

The recombinant TdaP5_gen_ combination vaccine has recently received approval of a specific pregnancy indication for use in pregnant women in Thailand. Whereas TdaP5_gen_ is currently available in a single dose prefilled syringe adapted to self-paid healthcare systems for pregnant women and other adolescents and adults in Thailand, lower-dose pertussis recombinant vaccines would offer a more affordable, highly effective option for pertussis vaccination of pregnant women in particular in LMICs. Since completion of this study, the lower-dose recombinant pertussis combination vaccine Tdap2_gen_ has been licensed in a multi-dose vial (Boostagen_RED_®) adapted to national immunization programs for vaccination of adults and adolescents aged 9 years and older in Thailand. Like the full-dose TdaP5_gen_ vaccine, the lower-dose recombinant Tdap2_gen_ vaccine has an approved specific pregnancy indication for use in pregnant women. Finally, full dose recombinant aP vaccines like aP5_gen_ and TdaP5_gen_ that induce a high and long-lasting antibody response, can be of high interest in countries where fast-waning immunity against pertussis is observed in populations primed with chemically detoxified acellular pertussis vaccines.

In summary, reduced dose investigational formulations of a licensed recombinant acellular pertussis vaccine containing PT_gen_ had an acceptable safety profile and were highly immunogenic in women of childbearing age. All formulations met minimum immunogenicity criteria and are now being evaluated for safety and immunogenicity in healthy pregnant women.

### CRediT authorship contribution statement

**Kulkanya Chokephaibulkit:** Supervision, Writing – review & editing. **Thanyawee Puthanakit:** Supervision, Writing – review & editing. **Niranjan Bhat:** Conceptualization, Methodology, Writing – review & editing. **Souad Mansouri:** Conceptualization, Methodology, Writing – review & editing, Supervision. **Yuxiao Tang:** Investigation, Writing – review & editing. **Keswadee Lapphra:** Investigation, Writing – review & editing. **Supattra Rungmaitree:** Investigation, Writing – review & editing. **Suvaporn Anugulruengkitt:** Investigation, Writing – review & editing. **Watsamon Jantarabenjakul:** Investigation, Writing – review & editing. **Indah Andi-Lolo:** Project administration, Writing – review & editing. **Renee Holt:** Project administration, Writing – review & editing. **Librada Fortuna:** Investigation, Writing – review & editing. **Chawanee Kerdsomboon:** Validation, Investigation, Writing – review & editing, Visualization. **Pailinrut Chinwangso:** Validation, Investigation, Writing – review & editing. **Ladda Suwitruengrit:** Investigation, Writing – review & editing. **Anita H.J. van den Biggelaar:** Writing – original draft, Writing – review & editing, Visualization. **Simonetta Viviani:** Conceptualization, Methodology, Writing – review & editing. **Pham Hong Thai:** Conceptualization, Methodology, Resources, Writing – review & editing. **Bruce L. Innis:** Conceptualization, Methodology, Writing – review & editing.

## Declaration of Competing Interest

The authors declare the following financial interests/personal relationships which may be considered as potential competing interests: [Librada Fortuna, Chawanee Kerdsomboon, Pailinrut Chinwangso, Ladda Suwitruengrit, Anita H.J. van den Biggelaar, Simonetta Viviani and Pham Hong Thai are employees of BioNet-Asia].
